# Risk factors for recollapse of new vertebral compression fractures after percutaneous kyphoplasty in geriatric patients: establishment of a nomogram

**DOI:** 10.1186/s12891-022-05409-3

**Published:** 2022-05-14

**Authors:** FuCheng Bian, GuangYu Bian, Li Zhao, Shuo Huang, JinHui Fang, YongSheng An

**Affiliations:** 1 Department of Endoscopic Diagnosis, Daqing Oilfield General Hospital, Heilongjiang 163000 Daqing, China; 2 Department of Orthopaedic, Chengde Medical University Affiliated Hospital, Chengde, 067000 Hebei China; 3 Department of Obstetrics, Daqing Oilfield General Hospital, Daqing, 163000 Heilongjiang China; 4 Department of Cardiac Surgery, Nanfang Hospital of Southern Medical University, Guangzhou, 510000 Guangdong China; 5 Department of Marketing and Tourism, Northeast Petroleum University, Daqing, 163000 Heilongjiang China

**Keywords:** Osteoporotic vertebral compression fracture, Percutaneous kyphoplasty, Risk factor, Nomogram

## Abstract

**Background:**

The main objective of this study was to investigate the risk factors for recollapse of new vertebral compression fractures (NVCFs) after percutaneous kyphoplasty (PKP) treatment for osteoporotic vertebral compression fracture (OVCF) and to construct a new nomogram model.

**Methods:**

We retrospectively analysed single-level OVCFs from January 2017 to June 2020, randomizing patients to a training set and a testing set. In the training set, independent risk factors for NVCFs in OVCF patients treated with PKP were obtained by univariate and multivariate regression analyses. These risk factors were then used as the basis for constructing a nomogram model. Finally, internal validation of the built model was performed in the testing set using the consistency index (C-index), receiver operating characteristic (ROC) curves, calibration curves and decision curve analysis (DCA).

**Results:**

In total, 371 patients were included in this study. NVCFs occurred in 21.7% of the training set patients, and multivariate regression analysis showed that a low Hounsfield unit (HU) value, cement leakage, and thoracolumbar (TL) junction fracture were independent risk factors for NVCF after PKP. The C-index was 0.81 (95% CI: 0.74–0.81), and the validation showed that the predicted values of the established model were in good agreement with the actual values.

**Conclusions:**

In this study, three independent risk factors were obtained by regression analysis. A nomogram model was constructed to guide clinical work and to make clinical decisions relatively accurately to prevent the occurrence of vertebral recollapse fractures.

## Background

The increasing ageing of the population has resulted in severe bone loss in elderly people, and osteoporotic vertebral compression fracture (OVCF) is one of the most common complications of osteoporosis, with an incidence of vertebral fracture exceeding 1/3 [1]. Vertebral fractures can occur with low-energy trauma (e.g., falls and violent coughing), resulting in acute or chronic low back pain, kyphosis, and prolonged bed rest and reducing patient survival [2, 3]. Approximately 40% of women who are over 50 years of age will experience an osteoporotic fracture in their lifetime [4].

Currently, percutaneous kyphoplasty (PKP) is widely used in clinical practice because of its minimally invasive and safe features, which can effectively relieve patients' back pain [5, 6]. However, complications associated with PKP include unsatisfactory reduction, cement leakage [7, 8], and adjacent vertebral fractures [9]. Seo et al. [10] suggested that after a fractured vertebral body is injected with bone cement, the increased stiffness of the strengthened vertebral body will lead to adjacent vertebral compression fractures (VCFs). However, Yi et al. [11] argued that there was no significant difference in the occurrence of postoperative new adjacent vertebral fractures in patients with cement-injected vertebral compared to patients treated conservatively. In addition, there are many studies on vertebral refractures and multiple risk factors that predispose patients to new vertebral compression fractures (NVCFs) [12, 13]. However, the sample sizes of these studies were small, and their clinical utility was not effective enough to translate into a convenient tool.

The risk of refracture of the vertebral body in patients treated with PKP/percutaneous vertebroplasty (PVP) has been widely considered, and relevant independent risk factors have been derived in previous studies [14, 15]. Unfortunately, few studies have visualized the obtained risk factors with data, so we constructed a nomogram model. Nomogram models are currently widely used, and a large number of them are applied in the diagnosis and prognosis of disease. A scientific and systematic approach was used to evaluate the probability of patients developing NVCFs postoperatively. By applying this model, surgeons can better prevent the recurrence of vertebral fractures after PKP and the unnecessary use of medical resources.

## Materials and methods

### Study subjects

This study reviewed patients who underwent PKP surgery for OVCFs from January 2017 to June 2020 and was approved by the ethics committee of Chengde Medical University Affiliated Hospital. The inclusion criteria were as follows: 1. a first single-level VCF in an elderly individual that was caused by a low-energy injury (e.g., fall, bending over) and was treated with PKP; 2. radiological examinations (MRI: T2-weighted imaging showing marked bone oedema of the fractured vertebral bodies) that confirmed the VCF; 3. back pain locally related to the VCF; 4. long-term local residence with no history of residency in other areas during the follow-up period; and 5. bilateral pedicles and posterior vertebral walls that were intact without fracture.

The exclusion criteria were as follows: 1. spinal cord or nerve root compression due to vertebral burst fractures; 2. infections, tumours, and systemic diseases; 3. steroid and long-term hormone use; and 4. presence of psychiatric abnormalities or language disorders.

All the patients included in this study were followed up for more than 1 year. Finally, the patients were divided into a training set (70%) and a testing set (30%) by using a randomized grouping method. The patients in the training set were used to construct the nomogram model, and their data were used to evaluate the value of this model in the testing set. All the patients were continuously given 0.25 µg/d calcitriol and D3 600 mg/d calcium after surgery. Additionally, after surgery, the patients received zoledronic acid once a year at a dose of 5 mg dissolved in 100 ml saline and were infused intravenously for at least 15 min. None of the patients received calcitonin or parathyroid drugs.

### Data collection

We reviewed previous literature and included risk factors that were likely to cause VCF after PKP. In addition, general patient characteristics and radiological parameters, including age, sex, BMI, kyphotic angle (preoperative and postoperative), volume of bone cement, leakage of cement (extending beyond the superior or inferior endplates), fracture level, HU value of CT, and bisphosphonate therapy, were collected.

### Surgical procedures

All the patients were treated with local anesthesia in the prone position. Two puncture needles were placed one-third anterior to the fractured vertebral body through bilateral pedicles under fluoroscopic guidance. Bilateral balloons were individually inflated to try to restore vertebral height, and then a "toothpaste-like" bone cement was injected. After the patients rested in bed for 6 h, a brace was worn for proper ambulation.

### Assessment

**Fig. 1:**
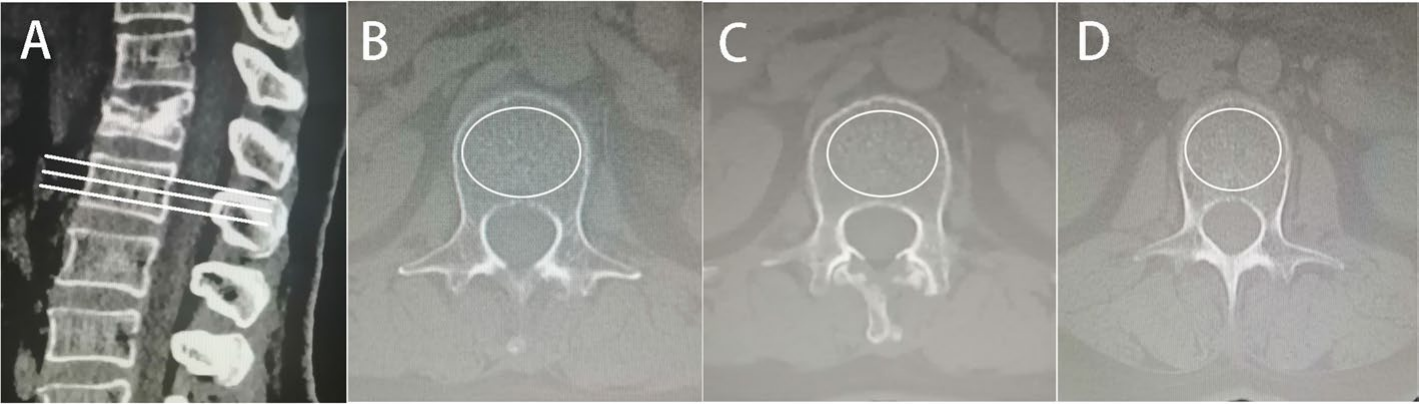
CT values were measured by PACS. **A** is a sagittal image of the lumbar spine with three tangents made on the measured vertebrae, corresponding to the three levels of (**B**), (**C**) and (**D**) in the axial position. The largest elliptical region of interest (ROI) containing only bone trabeculae was drawn in the axial position to obtain the average Hu value

A patient presented with acute low back pain, and the fractured vertebral showed a high signal on T2-weighted fat suppression imaging. The thoracolumbar (TL) junction was defined as T11 to L2. BMI was defined as the weight (kg) divided by the square of the height (m2). The angle between the inferior endplate of the superior vertebral of the fractured vertebral and the superior endplate of the inferior vertebral on the lateral radiograph was defined as the kyphotic angle. In this study, we used the HU value of the CT measurement [16]. The measurement was performed by using the picture archiving and communication system (PACS, version 3.6; YLZ Information Technology Co., Ltd; CHINA) of our hospital. First, three tangents (25%, 50% and 75%) were created for the measured vertebral in the sagittal position, and the largest elliptical region of interest (ROI) containing only bone trabeculae was drawn in the axial position to obtain the average CT value (Fig. 1). The mean CT value of the TL junction not including fractured vertebral was defined as the HU value of the patient [17]. An HU value of less than 100 was defined as osteoporosis, and an HU value of more than 100 and less than 150 was defined as osteopenia [18, 19].

### Statistical analysis

This study used SPSS 26 (IBM Corp., Armonk, NY, USA) for statistical analysis of the data. First, the training and testing sets were compared. Then, continuous variables were statistically analysed using Student's t test or the Mann–Whitney U test; the chi-square or Fisher's exact test was used for categorical variables. Second, the risk factors were obtained from univariate analysis, and multivariate regression analysis was used to obtain independent risk factors for the occurrence of NVCFs after PKP surgery. Finally, the independent risk factors were plotted into a predictive nomogram model using the "rms" package of R software (version 3.6.1).

Evaluation of the model can better show its practical value. The consistency index (C-index) evaluates the model's predictive ability, which ranges from 0 to 1, with moderate accuracy defined as 0.5–0.7, high accuracy defined as 0.7–0.9 and extreme accuracy defined as greater than 0.9. The calibration curve may reveal an excellent response in patients with NVCFs vs. predicted NVCFs. Decision curve analysis (DCA) was used to assess the utility of the nomogram model based on the probability of net benefits and thresholds.

## Results

A total of 393 patients were collected. According to the inclusion and exclusion criteria, 22 patients were excluded: 2 patients had language disorders, 8 patients lost to follow-up, and 12 patients had tumour diseases.

### Clinical characteristics

In all, 371 patients were included in this study. A total of 263 patients were used as the training set to construct the nomogram model, and 108 patients were used as the testing set to validate the model. The general characteristics of the patients in both groups are shown in Table 1. The average age of the patients in the training set was 72.48 ± 7.29 years and the average age of the patients in the testing set was 72.10 ± 7.54 years. There was no significant difference between the two groups (*P* > 0.05).

**Table 1:** Demographic characteristics of training set and testing set

Characteristics	Training set(263)	Testing set(108)	*P* value
Gender			0.06
Male	68	18	
Female	195	90	
Age(year)	72.48 ± 7.29	72.10 ± 7.54	0.65
BMI(Kg/m^2^)	23.16 ± 3.32	22.73 ± 3.08	0.25
HU value	81.13 ± 24.51	79.76 ± 27.05	0.63
Cement volume	4.74 ± 0.79	4.73 ± 0.83	0.87
Cement leakage			0.25
No	184	82	
Yes	79	26	
TL junction			0.38
No	99	46	
Yes	164	62	
Preoperative angle	18.52 ± 5.77	18.91 ± 6.65	0.64
Postoperative angle	12.35 ± 4.65	12.67 ± 4.89	0.55
Change angle	6.25 ± 4.03	6.25 ± 4.51	0.99
Bisphosphonates			0.24
No	83	41	
Yes	180	67	

*BMI* body mass index, *HU* Hounsfield unit, *TL* thoracolumbar

### Acquisition of independent risk factors and construction of nomogram models

**Fig. 2:**
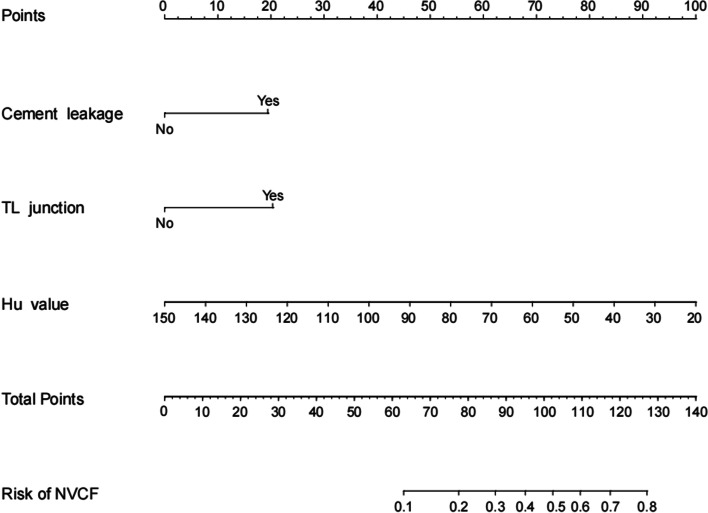
Nomogram for predicting NVCFs in patients with OVCFs after PKP operation. Each risk factor was assigned one point, which was summed to give a total number of points that corresponded to the probability of the hazard on the bottom row of the figure according to the total points

Among the patients in the training set, those in the NVCF and N-NVCF groups had statistically significant differences in Hu values, cement leakage, and TL segments (*P* < 0.05) (Table 2). These three factors were found to be risk factors for new fractures of the vertebral body by univariate regression analysis. Finally, a low Hu value (OR: 0.96, 95% CI: 0.94–0.97), cement leakage (OR: 2.96, 95% CI: 1.49–5.88) and TL segment (OR: 3.11, 95% CI: 1.41–6.89) were found to be independent risk factors for vertebral body refracture after PKP by multifactorial regression analysis (Table 3). A novel nomogram model was constructed based on these three independent risk factors (Fig. 2).

**Table 2:** Preoperative demographic characteristics in training set

Characteristics	NVCF(57)	N-NVCF(206)	*P* value
Gender			0.35
Male	12	56	
Female	45	150	
Age(year)	71.30 ± 7.58	72.81 ± 7.20	0.17
BMI(Kg/m^2^)	22.79 ± 3.51	23.26 ± 3.26	0.34
HU value	63.52 ± 24.20	86.01 ± 22.30	< 0.01
Cement volume	4.84 ± 0.83	4.71 ± 0.78	0.28
Cement leakage			< 0.01
No	27	157	
Yes	30	49	
TL junction			< 0.01
No	10	89	
Yes	47	117	
Preoperative angle	19.05 ± 6.26	18.47 ± 5.64	0.50
Postoperative angle	12.87 ± 5.06	12.20 ± 4.53	0.34
Change angle	6.19 ± 3.19	6.24 ± 4.25	0.88
Bisphosphonates			1.00
No	18	65	
Yes	39	141	

*BMI* body mass index, *HU* Hounsfield unit, *TL* thoracolumbar

**Table 3:** Univariate and multivariate logistic analysis of risk factors of NVCFs after OVCF

**Characteristics**	Univariate		Multivariate	
	*OR(95%CI)*	*P* value	*OR(95%CI)*	*P* value
Gender(female)	1.40(0.69–2.84)	0.35		
Age(year)	0.97(0.93–1.01)	0.17		
BMI(Kg/m^2^)	0.96(0.88–1.05)	0.34		
HU value	0.97(0.94–0.71)	< 0.01	0.96(0.94–0.97)	< 0.01
Cement volume	1.24(0.84–1.82)	0.28		
Cement leakage(yes)	3.56(1.93–6.56)	< 0.01	2.96(1.49–5.88)	< 0.01
TL junction(yes)	3.58(1.71–7.46)	< 0.01	3.11(1.41–6.89)	0.01
Preoperative angle	1.02(0.97–1.07)	0.49		
Postoperative angle	1.03(0.97–1.10)	0.34		
Change angle	1.00(0.92–1.07)	0.90		
Bisphosphonates(yes)	1.00(0.53–1.88)	1.00		

*NVCF* new vertebral compression fracture, *OVCF* osteoporotic vertebral compression fracture, *BMI* body mass index, *HU* Hounsfield unit, *TL* thoracolumbar

### Validation of the nomogram model

**Fig. 3:**
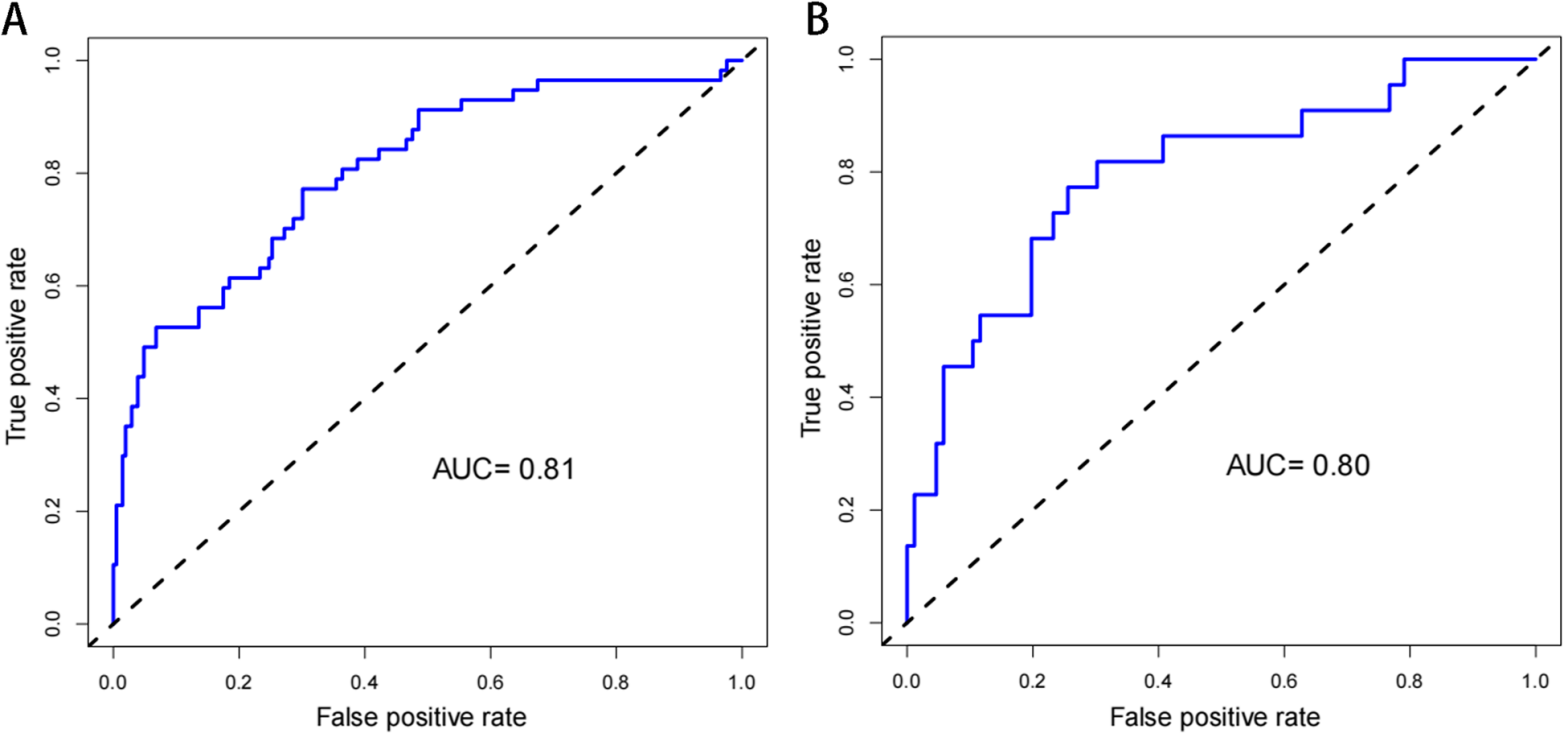
Comparison of the area under the receiver operating characteristic curve between nomogram-independent predictors in the training set (**A**) and the testing set (**B**)

**Fig. 4:**
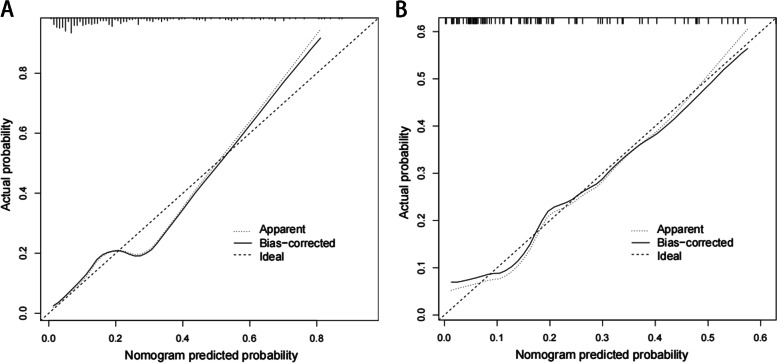
Comparison of calibration curves between the training set (**A**) and the testing set (**B**)

**Fig. 5:**
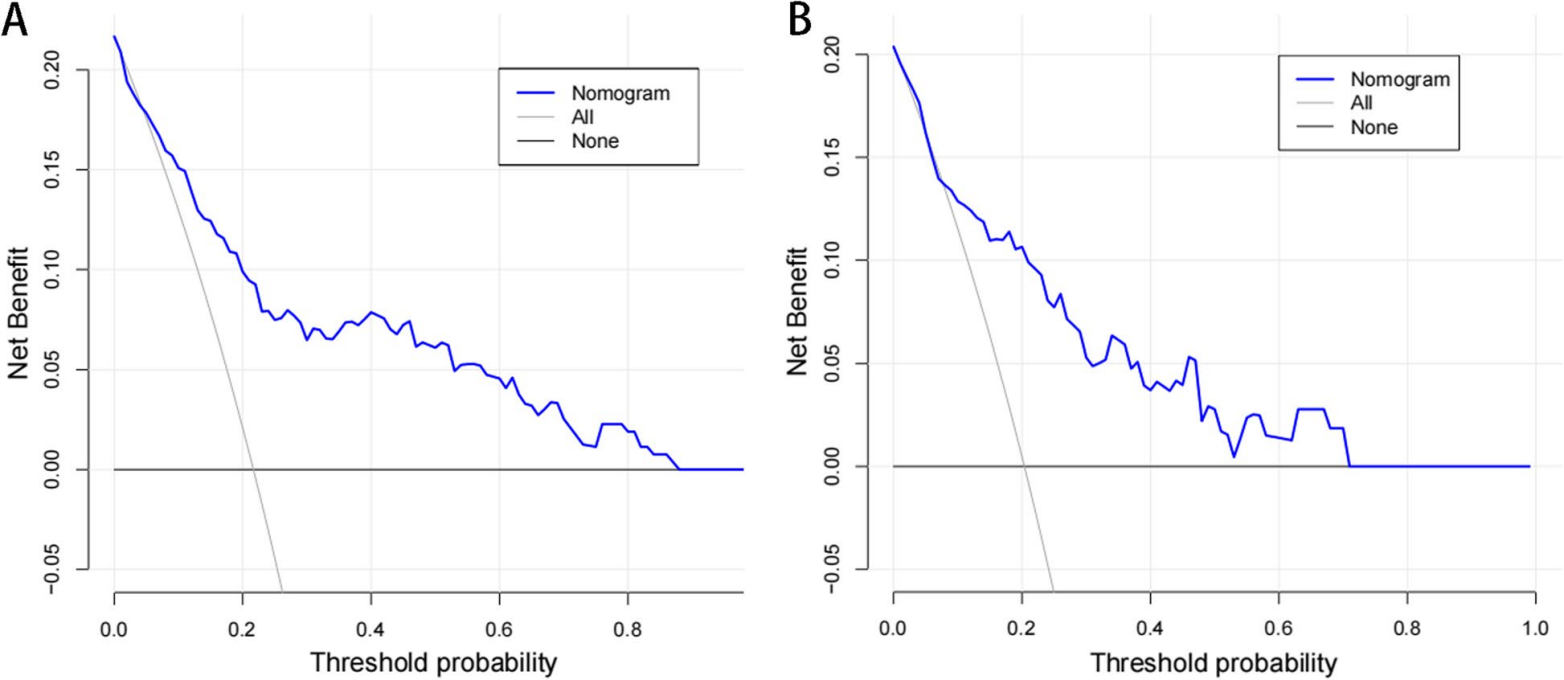
Comparison of decision curve analyses between the training set (**A**) and the testing set (**B**)

The constructed nomogram model was validated internally by the training set and testing sets. The C-index of this model was 0.81 (95% CI: 0.74–0.81), which shows that this model has high accuracy. In this study, receiver operating characteristic (ROC) curve were plotted, and areas under the ROC curve (AUC) of 0.81 and 0.80 were calculated for the training and testing sets, respectively (Fig. 3). The calibration curve results showed excellent agreement between the predicted probability and the actual probability of NVCFs (Fig. 4). DCA showed that this nomogram model could be an excellent prediction tool for NVCFs after PKP operation (Fig. 5).

### Use of the nomogram model

The constructed nomogram model has the characteristics of simplicity and convenience in clinical use. The scores corresponding to each independent risk factor were summed to obtain a total score, and the total score was then used to calculate the probability of NVCFs. For example, the HU value was 60 in a 75-year-old patient, the fracture segment was the TL junction, and there was cement leakage; therefore, the calculation is as follows, the HU score for this patient was 69, the TL junction score was 20, the cement leakage score was 19, and the total score was 69 + 20 + 19 = 118, which was equivalent to a 70% risk of NVCFs after PKP.

## Discussion

**Fig. 6:**
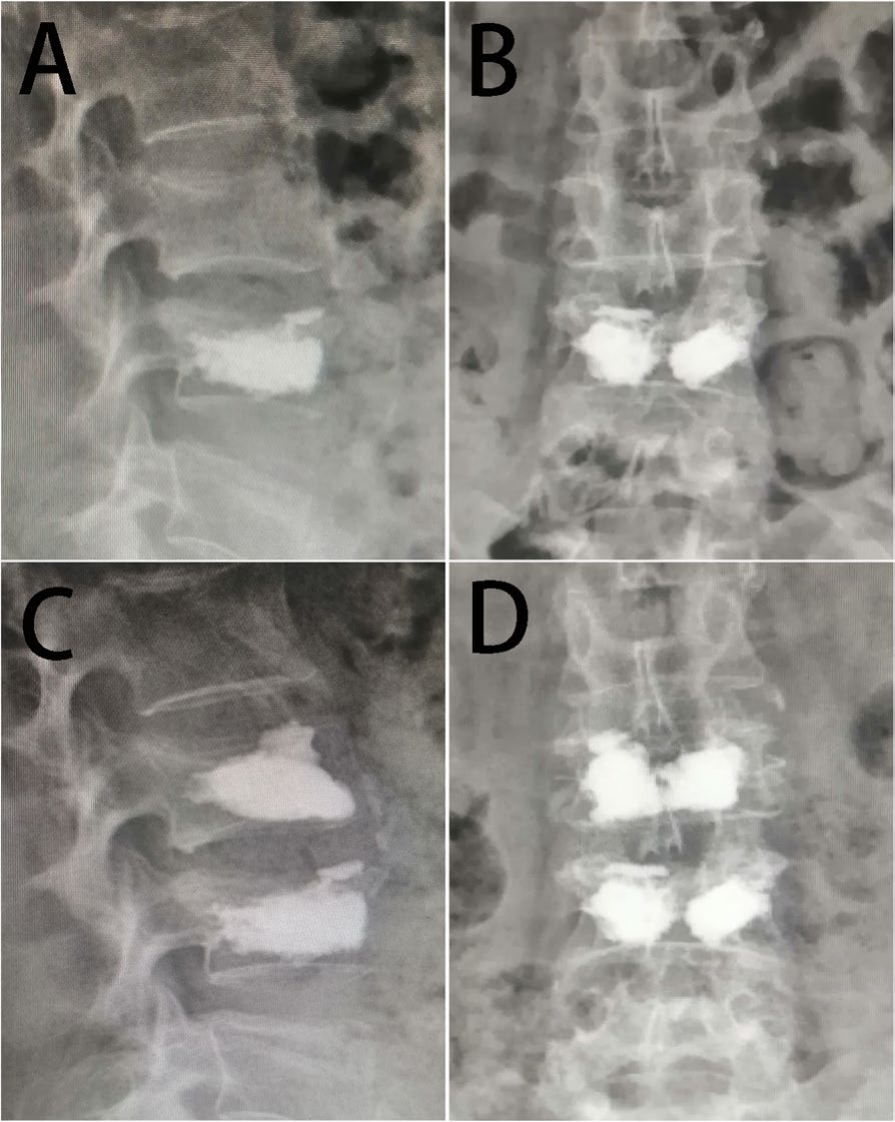
**A** and (**B**) are the lateral and anteroposterior X-rays after the first fracture operation; (**C**) and (**D**) are the lateral and positive X-rays after the second fracture

OVCFs are more likely to occur in elderly patients with osteoporosis, and patients undergoing minimally invasive surgical treatment can receive satisfactory results and an improved prognosis. However, fractured vertebral strengthened by bone cement are prone to postoperative NVCFs (Fig. 6). Some studies have shown that the probability of postoperative NVCFs in patients treated with PKP ranges from 6.5% to 34.8% [12, 20–22]. In this study, we found that a low HU value, bone cement leakage and TL junction fracture were independent risk factors by multivariate regression analysis for NVCFs after surgery. By obtaining clinically available risk factors, this study is the first to construct a nomogram model of postoperative refracture in PKP. Indeed, this study is different from previous investigations [13–15]. First, we developed a nomogram model, which was drawn from independent risk factors obtained by regression analysis, with the advantages of improved readability of the results and convenience of use. Second, a testing set was used in this study to verify the value of the established model. This nomogram model can help surgeons estimate the probability of postoperative refracture and develop individualized treatment or prevention plans for patients.

Bone mineral density (BMD) is usually measured by dual-energy X-ray absorptiometry (DXA) to obtain a T value. In clinical practice, DXA is not suitable for patients with spinal deformities, degenerative conditions or calcifications that would overestimate the T value [19]. Therefore, to improve the accuracy, some authors have started to search for new measurement methods. CT measurements are one of the standard tools used to diagnose fractures. Some scholars believe that CT (HU value) is an essential complementary means to indicate BMD, and it has been widely used in clinical work [17, 18, 23]. According to relevant studies, the HU value measured by CT can be obtained with high sensitivity and specificity [18, 24]. Several studies have shown that low BMD is not an independent risk factor for refracture, [21, 25] and the reason for this may be that the patients who develop OVCFs are elderly individuals whose lumbar degeneration is often severe, resulting in compensatory osteophytes, which reduce the accuracy of the T value. Therefore, instead of using the T value, the HU value was applied in this study. The results of this study show that the HU value (OR: 0.96) is an independent risk factor for NVCFs after surgery, which is consistent with previous studies [23].

Bone cement leakage is one of the common complications after PKP/PVP. In a follow-up study of 102 patients, Chen et al. [26] found that 14.7% of cement leakage cases occurred after PKP, and the integrity of the vertebral wall and the amount of cement injected were risk factors for cement leakage. A study reported that cement leakage was an independent risk factor for the occurrence of vertebral refracture after cement strengthening [17], which is similar to the results of this study. Li et al. [27] performed a multifactorial regression analysis of 403 patients in the training set and found that cement leakage, cement dispersion, and amount of cement were independent risk factors for vertebral refracture. Bone cement-related risk factors accounted for 3/6 this study, so bone cement has been a hot topic of related research. The intervertebral disc is the main cushioning device of the spine, and cement leakage into the disc can weaken the cushioning capacity, [28] making the vertebral more susceptible to NVCFs. By observing patients who underwent cement enhancement for OVCFs, Ahn et al. [29] attributed the refracture of adjacent vertebral to the direct pillar effect, which may be caused by the difference in strength after cement enhancement, and second, they attributed the refracture of nonadjacent segments to the dynamic hammer effect, which results in the difference in segmental mobility.

This study showed that TL junction fractures accounted for 56.0% of all fractures in the training set. TL junction (OR = 3.11) fractures were an independent risk factor for NVCFs. The TL junction is widely considered to be an excess region of biomechanical anatomy [30]. The TL junction is susceptible to NVCFs because it has a greater range of motion than other relatively inactive segments. A study demonstrated that older age is an independent risk factor for vertebral refracture [31]. However, Ji et al. [23] found that age and sex were not independent risk factors for the development of NVCFs after a multivariate regression analysis, which is consistent with the findings of this study. Li et al. [15] assessed 230 patients to analyse the risk factors for vertebral recollapse after undergoing PKP/PVP surgery. Regression analysis revealed no significant difference in the restoration of the Cobb angle(%) in the refracture group (40.38 ± 8.55) versus the non-refracture group (30.19 ± 10.24). This is consistent with our study.

Our nomogram model has strong practicability in the clinic. By applying it to patients with a first fracture, the risk of a second fracture can be estimated more accurately, and treatment can be personalized.

This study has some shortcomings. First, it was a retrospective analysis, all patients were from a single centre, and there was a lack of external data to verify the effectiveness of the model. Second, the follow-up time was short and should be extended. Finally, HU values measured by different CT devices may vary, and similarly, the same problem exists for DXA measurements, and standards for measurement need to be established.

## Conclusions

In this study, we retrospectively analysed patients who developed NVCFs after undergoing PKP treatment and found that a low HU value, cement leakage and TL junction fracture were independent risk factors for NVCFs. Therefore, a new nomogram model has been constructed by which surgeons can effectively assess the probability of new vertebral fractures after a patient's original OVCF and can intervene immediately after surgery to prevent refractures.

## Data Availability

The data that support the findings of this study are not openly available due to reasons of sensitivity of human data and are available from the corresponding author upon reasonable request.

## Abbreviations

NVCFs: New vertebral compression fractures
PKP: Percutaneous kyphoplasty
OVCF: Osteoporotic vertebral crush fracture
C index: Consistency index
ROC: Receiver operating characteristic
DCA: Decision curve analysis
HU: Hounsfield Units
TL: Thoracolumbar
N- NVCF: Non new vertebral compression fracture
BMD: Bone mineral density
DXA: Dual-energy x-ray absorptiometry
